# Prophylactic intracavitary treatment with interferon alpha increases interferon gamma production by peripheral blood mononuclear cells in patients with superficial transitional cell carcinoma of the bladder.

**DOI:** 10.1038/bjc.1997.315

**Published:** 1997

**Authors:** L. MoltÃ³, J. Carballido, L. Manzano, E. Reyes, C. Olivier, M. Alvarez-Mon

**Affiliations:** Department of Medicine, Hospital Universitario Principe de Asturias, University of AlcalÃ¡ de Henares, Madrid, Spain.

## Abstract

The immunomodulatory effect of prophylactic intravesical instillations of interferon alpha 2b (IFN-alpha-2b) on interferon gamma (IFN-gamma) and interleukin 4 (IL-4) production by peripheral blood mononuclear cells (PBMCs) from patients with superficial transitional cell carcinoma (STCC) of the bladder has been analysed. There were no significant differences in the production of IFN-gamma and IL-4 by PBMCs from untreated patients and healthy control subjects after 24 h of phytohaemagglutinin (PHA) stimulation. However, between 3 and 6 months after finishing the prophylactic intracavitary treatment with IFN-alpha-2b, PHA-stimulated PBMCs from patients with STCC of the bladder showed a significantly enhanced production of IFN-gamma and a significantly decreased production of IL-4. Both IFN-gamma and IL-4 returned to pretreatment levels 1 year after ending the treatment. In conclusion, prophylactic intravesical instillations of IFN-alpha-2b in patients with STCC of the bladder have an immunoregulatory effect on the production of IFN-gamma and IL-4 by PBMCs.


British Journal of Cancer (1997) 75(12), 1849–1853
© 1997 Cancer Research Campaign

### Prophylactic intracavitary treatment with interferon alpha increases interferon gamma production by peripheral blood mononuclear cells in patients with superficial transitional cell carcinoma of the bladder

**L Moltó[1], J Carballido[2], L Manzano[1], E Reyes[1], C Olivier[2] and M Alvarez-Mon[1]**

[1]Clinical Immunology Unit, Department of Medicine, Hospital Universitario 'Principe de Asturias', University of Alcalá de Henares, and [2]Department of Urology, Clinica 'Puerta de Hierro', Madrid, Spain

**Summary** The immunomodulatory effect of prophylactic intravesical instillations of interferon alpha 2b (IFN-α-2b) on interferon gamma (IFN-γ) and interleukin 4 (IL-4) production by peripheral blood mononuclear cells (PBMCs) from patients with superficial transitional cell carcinoma (STCC) of the bladder has been analysed. There were no significant differences in the production of IFN-γ and IL-4 by PBMCs from untreated patients and healthy control subjects after 24 h of phytohaemagglutinin (PHA) stimulation. However, between 3 and 6 months after finishing the prophylactic intracavitary treatment with IFN-α-2b, PHA-stimulated PBMCs from patients with STCC of the bladder showed a significantly enhanced production of IFN-γ and a significantly decreased production of IL-4. Both IFN-γ and IL-4 returned to pretreatment levels 1 year after ending the treatment. In conclusion, prophylactic intravesical instillations of IFN-α-2b in patients with STCC of the bladder have an immunoregulatory effect on the production of IFN-γ and IL-4 by PBMCs.

**Keywords:** superficial transitional cell carcinoma; interferon α; interferon γ; interleukin 4

Superficial transitional cell carcinoma (STCC) of the bladder is most often characterized by its high rate of recurrence following the initial endoscopic surgical resection, despite the absence of any identifiable remaining tumour (Torti and Lum, 1987). The heterogeneous natural history of STCC of the bladder (Lynch et al, 1991) after its endoscopic surgical resection provides a powerful rationale for the use of adjuvant intravesical therapy, in order to prevent the recurrence and progression of the disease. At present, the prophylactic use of intravesical chemotherapy in these patients continues to be of limited effectiveness, as it has failed to reduce significantly the rate of disease progression and long-term tumour recurrence (Lamm et al, 1992). Prophylactic immunotherapy of STCC, as exemplified by intravesical instillations with Bacillus Calmette–Guerin (BCG), has achieved positive results with a significant improvement in survival, presumably because of its ability to induce an effective immune response to tumours (Prescott et al, 1992; Kaempfer et al, 1996). However, the role of the immunotherapy in good/intermediate-risk superficial bladder cancer remains undefined. Other immunomodulators have also been investigated as prophylactic adjuvant treatment for STCC of the bladder (Jurincic et al, 1988; Glashan, 1990). The potential use of interferon alpha (IFN-α) as adjuvant prophylactic treatment of STCC of the bladder has also been investigated (Glashan, 1990). Comparative randomized clinical trials comparing BCG with IFN-α in the prophylactic treatment of patients with STCC of the bladder have not been performed. The mode of action of IFN-α in these patients remains partially defined. A direct effect of IFN-α on the tumour cells as well as immunomodulatory effects may be involved (Einat et al, 1985; Moltó et al, 1994; Singh et al, 1995). It has been shown that prophylactic intravesical instillations of IFN-α in patients with STCC are associated with in vivo immunomodulatory effects. Regulatory effects of intravesical instillations of IFN-α have been observed in PBMCs from patients with STCC of the bladder. These effects include an enhancement of NK-cell activity and a proliferative response to T-lymphocyte mitogens (Moltó et al, 1994, 1995).

Cytokines play an important role in the regulation of the function of the different immune cell populations. In this sense, several cytokines produced by T lymphocytes have stimulatory or inhibitory effects on the activation and proliferation of NK cells and cytotoxic T lymphocytes. It appears that different subsets of T-helper lymphocytes have different patterns of cytokine secretion as defined by the preferential secretion of either IFN-γ or IL-4, called $Th_1$ and $Th_2$ respectively (Seder & Paul, 1994). These cytokines show different regulatory effects on the activation of T lymphocytes and NK cells.

In this paper we investigate the effects of prophylactic intracavitary instillations of IFN-α-2b on the production of IFN-γ and IL-4 by phytohaemagglutinin (PHA)-stimulated PBMCs in patients with STCC of the bladder.

*Received 17 June 1996*
*Revised 13 January 1997*
*Accepted 14 January 1997*

*Correspondence to:* M Alvarez-Mon, Departamento de Medicina, Universidad de Alcalá de Henares, Carretera Madrid-Barcelona, km 33.600, 28871 Alcalá de Henares, Madrid, Spain

### PATIENTS AND METHODS

### Patients and treatments

Seventeen patients with histological proven transitional cell carcinoma of the bladder were analysed. The extent of tumour invasion was classified according to the tumour, node and metastasis

**Table 1** Patient and tumour characteristics

| | |
|---|---|
| No. of patients | 17 |
| No. of men/no. of women | 17/0 |
| Mean age ± SE at diagnosis | 66.2 ± 4.3 |
| Primary tumour / recurrent tumour | 12.5 |
| Solitary tumour / multiple tumours | 11/6 |
| Associated tumour in situ | 0 |
| Histological grade | |
| 1 | 0 |
| 2 | 9 |
| 3 | 8 |
| Histological stage | |
| pTA | 5 |
| pT1 | 12 |

staging system adopted by the International Union Against Cancer (Table 1). All the tumours were routinely completely resected with the muscle layer in each case, and random multiple biopsies were taken. None of the patients had received any treatment during the 6 months before the study. All the patients were studied before transurethral resection of the tumour (TUR), during the second month of a 3-month treatment with weekly intracavitary instillations of 50 × 10$^6$ IU of IFN-α-2b (Intron A, Schering-Plough, Kenilworth, NJ, USA) and 3, 6 and 12 months after ending treatment. Sixteen age- and sex-matched healthy individuals were also selected for the study as healthy control subjects. Blood samples were obtained before the surgical and anaesthetic procedures, and informed patient consent for the study was secured. The clinical evolution of the patients was analysed 1 year after finishing the prophylactic intravesical IFN-α-2b treatment. After this time, none of the five patients who were being treated for a recurrence of an earlier STCC had had a new recurrence, and nine of the patients who had no previous history of the disease had not had a recurrence. However, three patients who had had no previous history of the disease did have a recurrence during this time.

### Cell separation

PBMCs were obtained by density-gradient centrifugation (Ficoll-Hypaque) (Lymphoprep, Nyegaard, Oslo, Norway) and suspended in RPMI-1640 medium (Whitaker Bioproducts, Walkersville, MD, USA) containing 10% heat-inactivated fetal bovine serum (Biochrom, Berlin, Germany), L-glutamine (2 mM, Flow Lab., Irvine, UK), Hepes (0.5%, Flow Lab) and 1% penicillin–streptomycin (Difco Lab., Detroit, MI, USA). This will be referred to as complete medium. Next, cell viability was checked by trypan blue exclusion. After counting, cells were resuspended in complete medium.

### Cell cultures and measurement of cytokine productions

PBMCs (5 × 10$^6$ cells ml$^{-1}$) were cultured in complete medium on 24-macrowell plates (Costar, Cambridge, MA, USA) with phytohaemagglutinin M (PHA) (10 μg ml$^{-1}$, Difco Lab.) either alone or

**Figure 1** There were no significant differences in the production of IFN-γ (**A**) and IL-4 (**B**) by PBMCs (5 × 10$^6$ cells ml$^{-1}$) from untreated patients with STCC of the bladder ($n$ = 17) and healthy control subjects ($n$ = 16), after 24 h of PHA-stimulated culture ($P$ > 0.05). Results represent the median and interquartile ranges of duplicate samples performed in a solid-phase Intertest-γ ELISA kit and expressed as pg ml$^{-1}$. Horizontal bars within boxes show the median; boxes show the interquartile range from percentile 25 to percentile 75; vertical bars show the 90% confidence interval. □, Control subjects; ▨, patients

*© Cancer Research Campaign 1997*

**Figure 2** Production of IFN-γ by PHA-stimulated cultures of PBMCs from patients with STCC before and 3–6 months after finishing intracavitary IFN-α-2b instillations ($n = 8$) and from healthy control subjects ($n = 10$) was significantly enhanced in the presence of IFN-α-2b in the culture medium ($P < 0.05$) (**A**). However the addition of IFN-α-2b to PHA-cultures of PBMCs ($5 \times 10^6$ cells $ml^{-1}$) from the same untreated patients or from patients treated for 3–6 months with intracavitary IFN-α-2b ($n = 8$) or from healthy control subjects ($n = 10$) does not significantly increase IL-4 production after 24 h of culture ($P > 0.05$) (**B**). The results represent the median (horizontal bars) and interquartile ranges (from 25% to 75% for boxes and to 90% of confidence interval for vertical bars) of duplicate samples performed in a solid-phase Intertest-γ ELISA kit and expressed as pg $ml^{-1}$. *$P < 0.05$ compared with PHA alone. □, PHA; ▨, PHA + IFN-α-2b

**Figure 3** IFN-γ production by PHA-stimulated cultures of PBMCs from patients with STCC of the bladder treated with intracavitary IFN-α-2b instillations ($n = 13$) was significantly enhanced 3–6 months after finishing the treatment compared with the levels found before treatment ($P < 0.05$), and declined thereafter ($P > 0.05$). However, the production of IL-4 by PHA-stimulated cultures from patients decreased significantly 3 and 6 months after finishing the treatment compared with the levels found before and during treatment ($P < 0.05$). PBMCs ($5 \times 10^6$ cells $ml^{-1}$) were cultured in the presence of the indicated amounts of PHA for 24 h, and then the supernatant was harvested, filtered and stored at –40°C until use. The results represent the median (horizontal bars within boxes) and interquartile range (from 25% to 75% for boxes and to 90% of confidence interval for vertical bars) of duplicate samples performed in a solid-phase Intertest-γ ELISA kit and expressed as pg $ml^{-1}$. *$P < 0.05$ compared with pretreatment values. □, IFN-γ; ▨, IL-4

in the presence of IFN-α-2b (1000 IU $ml^{-1}$). Cultures were incubated for 24 h at 37°C in a humid atmosphere containing 5% carbon dioxide. Supernatants were harvested after incubation, sterilized by filtration through an 0.22-μm filter (Millipore, Bedford, CA, USA), aliquoted and quickly stored at –40°C until measurement. Concentrations of cytokines were assayed using commercially available IFN-γ (Endogen, Boston, MA, USA) and IL-4 (Genzyme, Cambridge, MA, USA) ELISA kits. The results are expressed as pg $ml^{-1}$. The detection limit of the IFN-γ and IL-4 test kits are 100 and 45 pg $ml^{-1}$ respectively.

### Staining of cells and flow cytometry analysis

For immunofluorescence staining, PBMCs were incubated with combinations of fluorescein (FITC, green) and phycoerythrin (PE, red)-labelled monoclonal antibodies (MAbs). Control studies involving unstained cells and cells incubated with isotype-matched irrevelant FITC- and PE-labelled MAbs were performed with each experiment. The following two- and one-colour MAbs were used to identify PBMC populations: leucoGATE (anti-leucocyte FITC/Anti-Leu-M3 PE) (CD45/CD14), control ($IgG_1$ FITC/$IgG_2$ PE), Simultest anti-Leu-4 (FITC) + anti-Leu-11c+19 (PE)(CD3/CD16–56), anti-Leu-3 (FITC) + anti-Leu-2a (PE)(CD4/CD8) and anti-Leu-12 (FITC)(CD19). All MAbs were obtained from Beckton-Dickinson (Mountain View, CA, USA). Acquisition and analysis for two-colour immunofluorescence procedures were carried out with a FACScan flow cytometer using Lysis II software.

### Statistical analysis

To analyse the results, data from the groups were compared with the unpaired Mann–Whitney *U*-test. For paired comparisons of data from the same group, determinations were made using the Wilcoxon matched-pairs sign test. A *P*-value of less than 0.05 was considered to indicate a significant difference between groups. Cytokine levels are given as the median and interquartile range.

### RESULTS

### Significative increase of IFN-γ and significative decrease of IL-4 production by PHA-stimulated PBMCs from patients with STCC of the bladder after intravesical prophylactic treatment with IFN-α-2b

The first step was to study IFN-γ production by PHA-stimulated PBMCs from patients with STCC of the bladder before the beginning of treatment, and from healthy control subjects. There were

© Cancer Research Campaign 1997

**Table 2** Phenotypical distribution of PBMCs from patients with STCC of the bladder before and after prophylactic treatment with intracavitary IFN-α-2b instillations ($n$ = 8) and from healthy control subjects ($n$ = 12)

| | | STCC patients | |
|---|---|---|---|
| **PBMCs** | **Control subjects** | **Before treatment** | **3 months after treatment** |
| $CD3^+$ | 70 (61–77) | 70 (63–73) | 70 (62–79) |
| $CD16^+/56^+$ | 26 (16–33) | 28 (23–31) | 33 (25–40) |
| $CD19^+$ | 9 (5–13) | 10 (6–14) | 11 (9–13) |
| $CD4^+$ | 42 (37–46) | 34 (31–38) | 30 (27–35)[a] |
| $CD8^+$ | 31 (28–36) | 33 (29–37) | 38 (33–41) |
| Ratio CD4/CD8 | 1.68 (1.5–1.7) | 1.19 (0.6–1.3) | 0.8 (0.6–1.4)[b] |

Results are expressed as median and interquartile range. [a]$P < 0.05$ vs $CD4^+$ of healthy control subjects. [b]$P < 0.05$ vs the ratio of $CD4^+/CD8^+$ PBMCs of control subjects.

no statistically significant differences in PBMC production of IFN-γ between the patients with STCC of the bladder and the healthy control subjects ($P > 0.05$) (Figure 1A).

The effect of IFN-α-2b in the culture medium on the production of IFN-γ by PHA-stimulated PBMCs from patients and healthy control subjects was also studied. As shown in Figure 2A, the production of IFN-γ by PHA-stimulated PBMCs from STCC patients, studied before treatment and 3–6 months after finishing the 3-month treatment with intracavitary IFN-α-2b instillations, and from healthy control subjects, was significantly enhanced in the presence of IFN-α-2b in the culture medium ($P < 0.05$).

The effects of prophylactic IFN-α-2b intravesical instillations on the production of IFN-γ by PHA-stimulated PBMCs in patients were analysed several times during the first year of follow-up. As shown in Figure 3, there was no increase in the production of IFN-γ by PHA-stimulated PBMCs from patients during the second month of treatment ($P > 0.05$). The increase in IFN-γ production by PHA-stimulated PBMCs from patients analysed between 3 and 6 months after finishing the treatment, with respect to that found before initiating the treatment and during the 3 months of intracavitary IFN-α-2b instillations, was significant ($P < 0.05$). There were no significant differences in IFN-γ production by PHA-stimulated PBMCs from patients before the treatment and 1 year after finishing the intracavitary instillations with IFN-α-2b ($P > 0.05$).

IL-4 production was studied at the same time and under the same conditions as IFN-γ production. There were no statistically significant differences in the production of IL-4 by PBMCs between untreated STCC patients and healthy control subjects ($P > 0.05$) (Figure 1B).

The effect of IFN-α-2b on the production of IL-4 by PHA-stimulated PBMCs from patients with STCC of the bladder, before treatment and 3–6 months after finishing the intracavitary IFN-α-2b instillations and from healthy control subjects was examined. Figure 2B, shows that in both healthy control subjects and patients the exogenous addition of IFN-α-2b to the culture medium of PHA-stimulated PBMCs did not significantly modify the production of IL-4 with respect to that achieved in its absence ($P > 0.05$).

The effects of the prophylactic intravesical instillation of IFN-α-2b on the production of IL-4 by PHA-stimulated PBMCs from the patients during a year's follow-up was also studied. As shown in Figure 3, there were no significant differences in IL-4 production by PHA-stimulated PBMCs from patients measured during the second month of treatment with intracavitary instillations of IFN-α-2b, and that found before treatment ($P > 0.05$). However, there was a significant decrease in IL-4 production by PHA-stimulated PBMCs from patients when analysed between 3 and 6 months after finishing the treatment, with respect to that found before initiating the treatment ($P < 0.05$). There were no significant differences between IL-4 production by PHA-stimulated PBMCs from patients before treatment and that after 1 year of finishing the intracavitary instillations with IFN-α-2b ($P > 0.05$) (Figure 3).

IFN-γ and IL-4 production by PHA-activated PBMCs was analysed in six healthy control subjects at the beginning and at the end of a 6-month period. No significant differences were found in the cytokine production between these samples ($P > 0.05$) (data not shown).

As shown in Table 2, there were no significant differences in the percentages of cells that express the antigens CD3, CD16/56, CD19, CD4 and CD8 in PBMCs from untreated patients with STCC of the bladder and healthy control subjects. However, 3 and 6 months after finishing the 3-months intracavitary instillations with IFN-α-2b, the percentage of $CD4^+$ lymphocytes and the $CD4^+/CD8^+$ ratio in PBMCs from STCC patients were significantly decreased compared with those found in healthy control subjects ($P < 0.05$), but there were no significant differences with respect to the values found in these patients before their intracavitary treatment ($P > 0.05$).

### DISCUSSION

The cytokines IFN-γ and IL-4 play a pivotal role in the regulation of the immune response induced by antigenic stimulation. IFN-γ is mainly produced by T lymphocytes and NK cells. It is known that IFN-γ regulates the activation of putative anti-tumour effector cells, including macrophages, NK cells and cytotoxic T lymphocytes (Kasahara et al, 1983; Murakata et al, 1985). IFN-γ production by PBMCs is frequently impaired in patients with advanced neoplasms (Ikemoto et al, 1990). IL-4 is mainly produced by T lymphocytes (Hayakawa et al, 1991). IL-4 is involved in the modulation of activation and proliferation of cytotoxic T lymphocytes, and there are controversial reports about its effects on NK cells (Hayakawa et al, 1991; Spits et al, 1988; Higuchi et al, 1989). There is increasing evidence that different subsets of T lymphocytes have different patterns of cytokine secretion, as defined according to the preferential secretion of IFN-γ or IL-4, called $Th_1$ and $Th_2$ respectively. It appears that both T-lymphocyte subsets negatively cross-regulate each other (Heinzel et al, 1989).

Our results show that PBMCs from untreated patients with STCC of the bladder have a normal production of IFN-γ and IL-4.

 © *Cancer Research Campaign 1997*

Intracavitary prophylactic treatment with IFN-α-2b in these patients is associated with a significant modification of the pattern of PBMC secretion of these two cytokines. The increased IFN-γ production parallels a decreased IL-4 production by PBMCs from these patients. This effect is mainly observed 3–6 months after finishing the intracavitary treatment with IFN-α-2b and has virtually disappeared after 12 months of follow-up. These modifications in the pattern of IFN-γ and IL-4 secretion by PBMCs found in patients with STCC of the bladder prophylactically treated with intracavitary IFN-α-2b instillations suggest that the induction of a bias towards the preferential use and/or activation of the T-lymphocyte subset that produces IFN-γ in these patients. The $Th_1$ subset appears to be mainly implicated in the regulation of immune responses involving cytotoxic T lymphocytes and NK cells. It seems that the modification in the pattern of cytokine secretion by PBMCs from STCC patients treated with intracavitary IFN-α-2b, might be involved in the described activation of NK cells and T lymphocytes found in the peripheral blood of these patients (Moltó et al, 1994, 1995).

Our findings clearly show that prophylactic intracavitary treatment with IFN-α-2b in patients with STCC of the bladder not only has a local effect on the bladder wall, but also has a systemic immunomodulatory effect on the T-lymphocyte compartment, with a marked functional consequence on the pattern of lymphokine secretion. The mechanism by which a locally administered cytokine can induce an extended systemic effect is as yet unknown. Perhaps the intravesical instillations of IFN-α induce a local immune response in the bladder wall that has a delayed systemic consequence. The systemic effects of intravesical instillations with IFN-α might be explained by the recirculation of locally activated T lymphocytes and/or as a consequence of the generalized functional interactions found in the immune system.

According to the known biological effects of IFN-α, the mechanisms of action of the intravesical instillations of IFN-α as a prophylactic anti-tumoral treatment might be multiple (Einat et al, 1985; Singh et al, 1995). The potential clinical significance of these findings has to be studied. The analysis of the immunoregulatory effects of IFN-α may be a useful tool in the optimization of this therapeutical modality in the prophylaxis of the recurrences of STCC of the bladder. Our data clearly show that the prophylactic intracavitary treatment of patients with STCC of the bladder with IFN-α-2b has a transient systemic immunomodulatory effect. It might be possible to suggest that the maintenance of the immune system activation could improve the clinical results obtained with prophylactic instillations of IFN-α in patients with STCC of the bladder. However, analysis of the potential clinical significance of the immunomodulatory effects of the prophylactic intracavitary instillations of IFN-α-2b in patients with STCC of the bladder requires further studies.

### ACKNOWLEDGEMENTS

The authors wish to thank Cesar Gonzalez and Jorge Cardona for their expert technical help, María José Sanchez and Carmen Martin for their secretarial assistance and Carol F. Warren of the Instituto de Ciencias de la Educación of UAH for her linguistic assistance. This work was partially supported by a grant from the Comisión Interministerial de Ciencia y Tecnología, SAF93-0925-C02-02 and from the Comunidad Autonoma de Madrid, SAL C265/91.

### REFERENCES

Einat M, Resnitzky D and Kimchi A (1985) Close link between reduction of c-*myc* expression by interferon and $G_0/G_1$ arrest. *Nature* **313**: 597–600

Glashan RW (1990) A randomized controlled study of intravesical alpha-2b-interferon in carcinoma *in situ* of the bladder. *J Urol* **144**: 658–661

Hayakawa K, Salmeron MA, Kornbluth J, Bucana C and Itoh K (1991) The role of interleukin-4 in proliferation and differentiation of human natural killer cells. Study of an interleukin-4-dependent *versus* an interleukin-2-dependent natural killer cell clone. *J Immunol* **146**: 2453–2460

Heinzel FP, Sadick MD, Holaday BJ, Coffman, RL and Locksley RM (1989). Reciprocal expression of interferon gamma or interleukin 4 during the resolution or progression of murine leishmaniasis. Evidence for expansion of distinct helper T cell subsets. *J Exp Med* **169**: 59–72

Higuchi CM, Thompson JA, Lindgren CG, Gillis S, Widmer MB, Kern DE and Pefer A (1989) Induction of lymphokine-activated killer activity by interleukin 4 in human lymphocytes pretreated with interleukin 2 *in vivo* or *in vitro*. *Cancer Res* **46**: 6487–6492

Ikemoto S, Kishimoto T, Wada S, Nishio S and Maekawa M (1990) Clinical studies on cell-mediated immunity in patients with urinary bladder carcinoma: blastogenic response, interleukin-2 production and interferon-γ production of lymphocytes. *Br J Urol* **65**: 333–338

Kaempfer R, Gerez L, Farbstein H, Madar L, Hirschman O, Nussinovich R and Shapiro A (1996) Prediction of response to treatment in superficial bladder carcinoma through pattern of interleukin-2 gene expression. *J Clin Oncol* **14**: 1778–1786

Kasahara T, Hooks JJ, Dougherty SF and Oppenheim JJ (1983) Interleukin 2-mediated immune interferon ($IFN_γ$) production by human T cells and T-cell subsets. *J Immunol* **130**: 1784–1789

Jurincic CD, Engelmann U, Gasch J and Klippel KF (1988) Immunotherapy in bladder cancer with keyhole-limpet hemocyanin: a randomized study. *J Urol* **139**: 723–726

Lamm DL, Griffith G, Pettit LL and Nseyo UO (1992) Current perspectives in diagnosis and treatment of superficial bladder cancer. *Urology* **39**: 301–308

Lynch CF, Platz CE, Jones MP and Gazzaniga JM (1991) Cancer registry problems in classifying bladder cancer. *J Natl Cancer Inst* **83**: 429–433

Molto L, Alvarez-Mon M, Carballido J, Manzano L, Guillen C, Prieto A, Olivier C and Rodriguez-Zapata M (1994) Intracavitary prophylactic treatment with interferon alpha 2b of patients with superficial bladder cancer is associated with a systemic T-cell activation. *Br J Cancer* **70**: 1247–1251

Molto L, Alvarez-Mon M, Carballido J, Olivier C and Manzano L (1995) Use of intracavitary interferon-alpha-2b in the prophylactic treatment of patients with superficial bladder cancer. *Cancer* **75**: 2720–2726

Murakata T, Semba V, Shibuya Y, Kuwano K, Akagi M and Arai S (1985) Induction of Interferon-γ production by human natural killer cells stimulated by hydrogen peroxide. *J Immunol* **134**: 2449–2455

Prescott S, James K, Hargreave TB, Chisholm GD and Smyth JF (1992) Intravesical Evans strain BCG therapy: quantitative immunohistochemical analysis of the immune response within the bladder wall. *J Urol* **147**: 1636–1642

Seder RA and Paul WE (1994) Acquisition of lymphokine-producing phenotype by CD4+ T cells. *Annu Rev Immunol* **12**: 635–673

Singh RK, Gutman M, Bucana CD, Sanchez R, Llansa N and Fidler I (1995) Interferons α and β down-regulate the expression of basic fibroblast growth factor in human carcinomas. *Proc Natl Acad Sci USA* **92**: 4562–4566

Spits H, Yssel H, Paliard X, Kastelein R, Figdor C and De Vries J (1988) Interleukin-4 inhibits interleukin-2-mediated induction of human lymphokine-activated killer cells, but not the generation of antigen-specific cytotoxic T lymphocytes in mixed leukocyte cultures. *J Immunol* **141**: 29–36

Torti FM and Lum BL (1987) Superficial bladder cancer. Risk of recurrence and potential role for interferon therapy. *Cancer* **59**: 613–616

© Cancer Research Campaign 1997


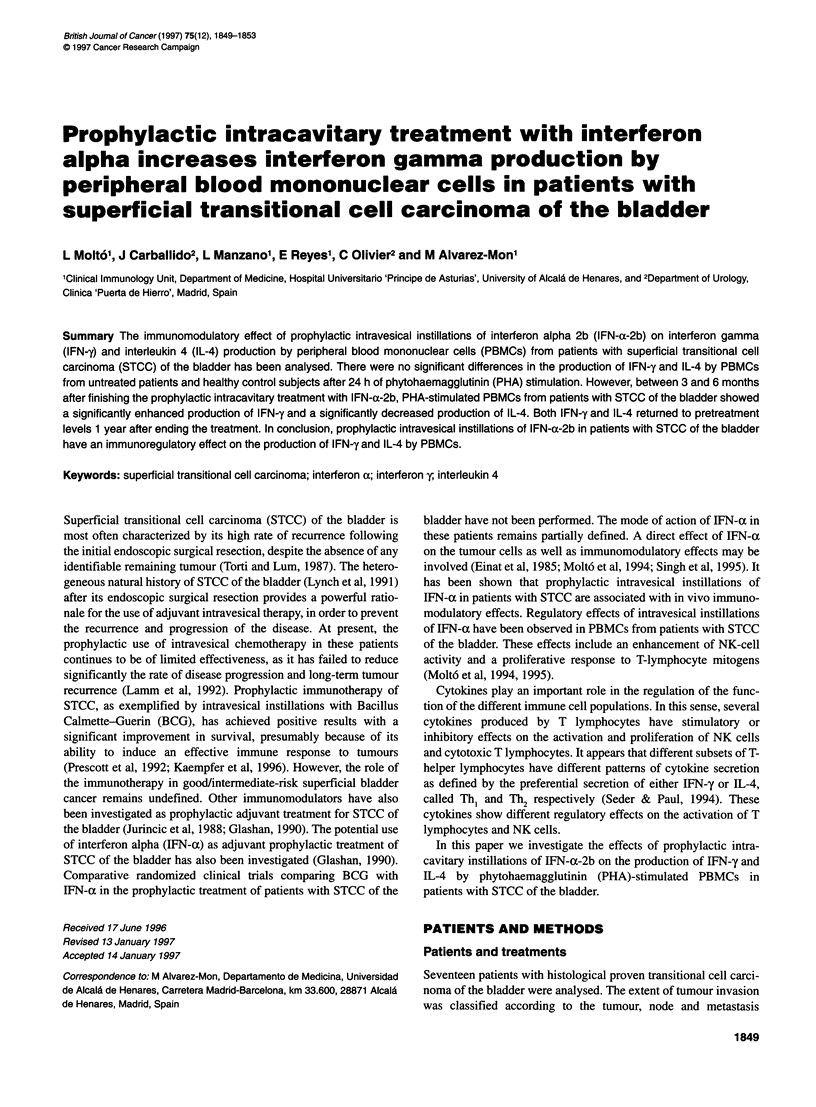

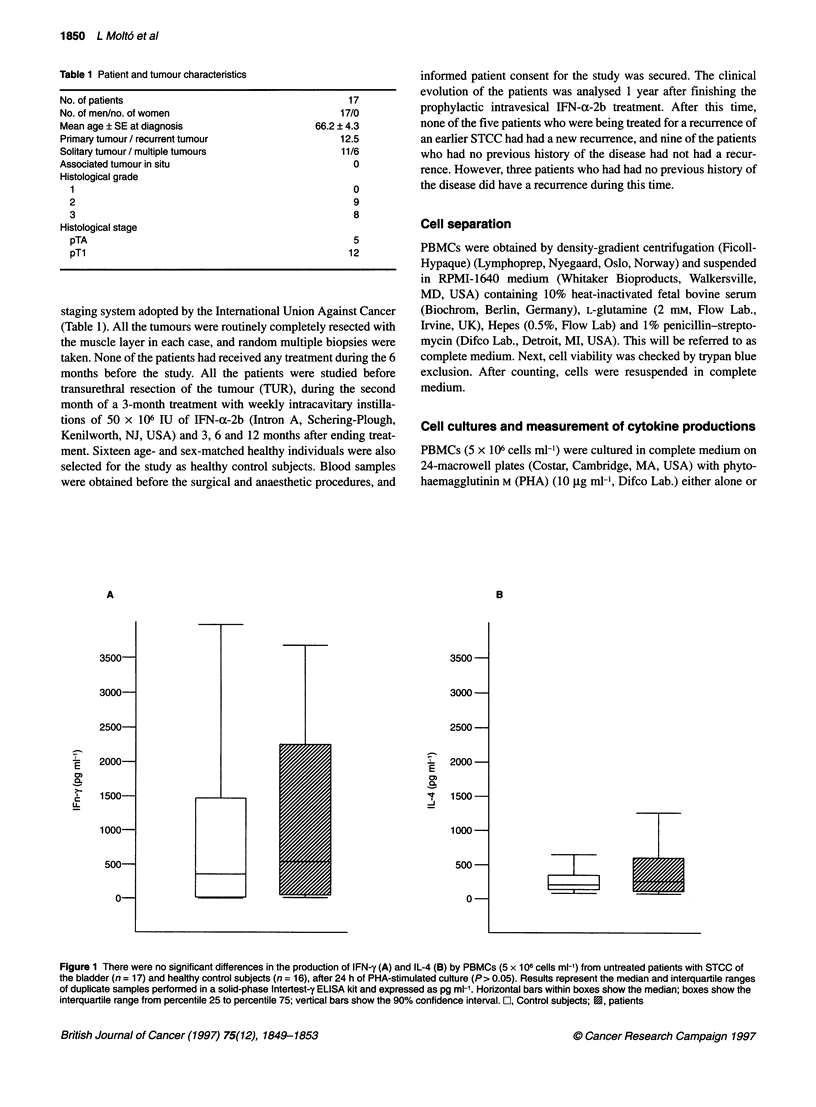

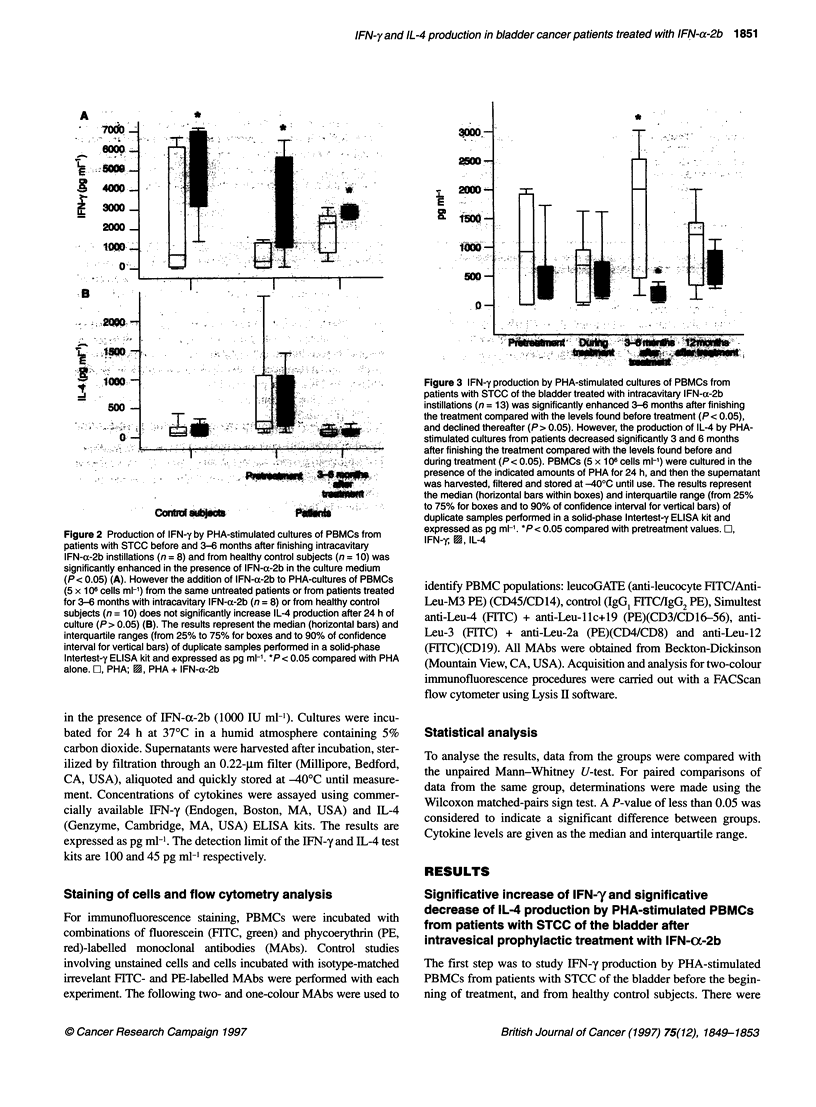

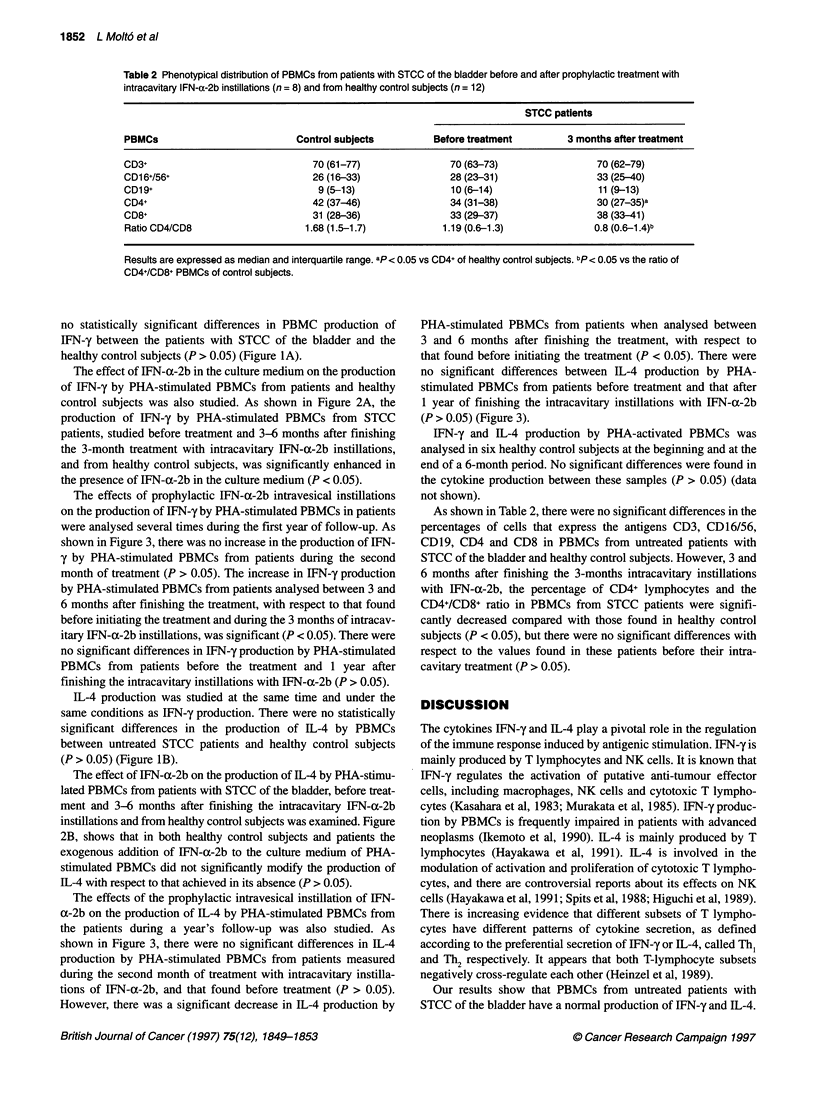

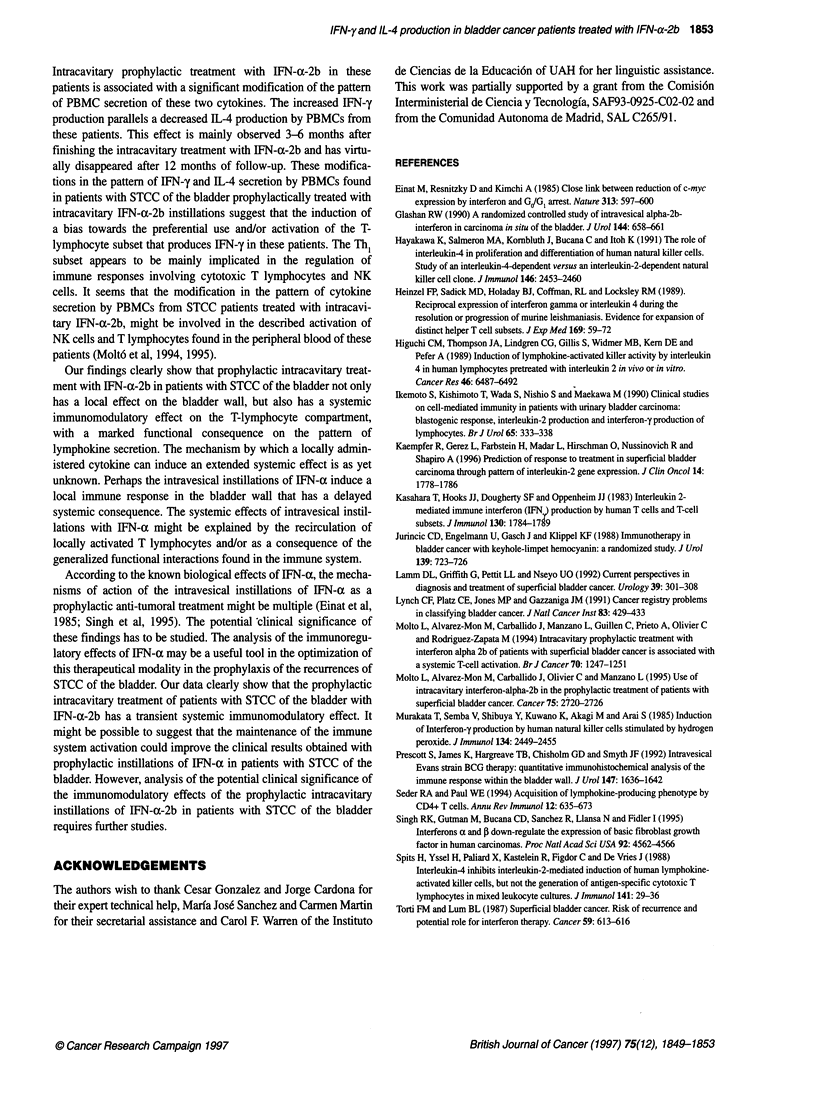


## References

[OCR_00588] Einat M., Resnitzky D., Kimchi A. (1985). Close link between reduction of c-myc expression by interferon and, G0/G1 arrest.. Nature.

[OCR_00592] Glashan R. W. (1990). A randomized controlled study of intravesical alpha-2b-interferon in carcinoma in situ of the bladder.. J Urol.

[OCR_00596] Hayakawa K., Salmeron M. A., Kornbluth J., Bucana C., Itoh K. (1991). The role of IL-4 in proliferation and differentiation of human natural killer cells. Study of an IL-4-dependent versus an IL-2-dependent natural killer cell clone.. J Immunol.

[OCR_00603] Heinzel F. P., Sadick M. D., Holaday B. J., Coffman R. L., Locksley R. M. (1989). Reciprocal expression of interferon gamma or interleukin 4 during the resolution or progression of murine leishmaniasis. Evidence for expansion of distinct helper T cell subsets.. J Exp Med.

[OCR_00610] Higuchi C. M., Thompson J. A., Lindgren C. G., Gillis S., Widmer M. B., Kern D. E., Fefer A. (1989). Induction of lymphokine-activated killer activity by interleukin 4 in human lymphocytes preactivated by interleukin 2 in vivo or in vitro.. Cancer Res.

[OCR_00616] Ikemoto S., Kishimoto T., Wada S., Nishio S., Maekawa M. (1990). Clinical studies on cell-mediated immunity in patients with urinary bladder carcinoma: blastogenic response, interleukin-2 production and interferon-gamma production of lymphocytes.. Br J Urol.

[OCR_00635] Jurincic C. D., Engelmann U., Gasch J., Klippel K. F. (1988). Immunotherapy in bladder cancer with keyhole-limpet hemocyanin: a randomized study.. J Urol.

[OCR_00623] Kaempfer R., Gerez L., Farbstein H., Madar L., Hirschman O., Nussinovich R., Shapiro A. (1996). Prediction of response to treatment in superficial bladder carcinoma through pattern of interleukin-2 gene expression.. J Clin Oncol.

[OCR_00630] Kasahara T., Hooks J. J., Dougherty S. F., Oppenheim J. J. (1983). Interleukin 2-mediated immune interferon (IFN-gamma) production by human T cells and T cell subsets.. J Immunol.

[OCR_00640] Lamm D. L., Griffith G., Pettit L. L., Nseyo U. O. (1992). Current perspectives on diagnosis and treatment of superficial bladder cancer.. Urology.

[OCR_00644] Lynch C. F., Platz C. E., Jones M. P., Gazzaniga J. M. (1991). Cancer registry problems in classifying invasive bladder cancer.. J Natl Cancer Inst.

[OCR_00648] Molto L., Alvarez-Mon M., Carballido J., Manzano L., Guillen C., Prieto A., Olivier C., Rodriguez-Zapata M. (1994). Intracavitary prophylactic treatment with interferon alpha 2b of patients with superficial bladder cancer is associated with a systemic T-cell activation.. Br J Cancer.

[OCR_00655] Moltó L., Alvarez-Mon M., Carballido J., Olivier C., Gimeno F., Manzano L. (1995). Use of intracavitary interferon-alpha-2b in the prophylactic treatment of patients with superficial bladder cancer.. Cancer.

[OCR_00660] Munakata T., Semba U., Shibuya Y., Kuwano K., Akagi M., Arai S. (1985). Induction of interferon-gamma production by human natural killer cells stimulated by hydrogen peroxide.. J Immunol.

[OCR_00665] Prescott S., James K., Hargreave T. B., Chisholm G. D., Smyth J. F. (1992). Intravesical Evans strain BCG therapy: quantitative immunohistochemical analysis of the immune response within the bladder wall.. J Urol.

[OCR_00670] Seder R. A., Paul W. E. (1994). Acquisition of lymphokine-producing phenotype by CD4+ T cells.. Annu Rev Immunol.

[OCR_00674] Singh R. K., Gutman M., Bucana C. D., Sanchez R., Llansa N., Fidler I. J. (1995). Interferons alpha and beta down-regulate the expression of basic fibroblast growth factor in human carcinomas.. Proc Natl Acad Sci U S A.

[OCR_00678] Spits H., Yssel H., Paliard X., Kastelein R., Figdor C., de Vries J. E. (1988). IL-4 inhibits IL-2-mediated induction of human lymphokine-activated killer cells, but not the generation of antigen-specific cytotoxic T lymphocytes in mixed leukocyte cultures.. J Immunol.

[OCR_00684] Torti F. M., Lum B. L. (1987). Superficial bladder cancer. Risk of recurrence and potential role for interferon therapy.. Cancer.

